# Microalgae Flocculation: Assessment of Extraction Yields and Biological Activity

**DOI:** 10.3390/ijms251910238

**Published:** 2024-09-24

**Authors:** Paola Imbimbo, Alfonso Ferrara, Enrica Giustino, Davide Liberti, Daria Maria Monti

**Affiliations:** 1 Department of Chemical Sciences, University of Naples Federico II, via Cinthia 4, 80126 Naples, Italy; paola.imbimbo@unina.it (P.I.); alfonso.ferrara@unina.it (A.F.); enrica.giustino@unina.it (E.G.); 2 Centre of Marine Sciences, University of Algarve, Campus de Gambelas, 8005-139 Faro, Portugal; dliberti@ualg.pt

**Keywords:** microalgae, flocculation, harvesting, green extraction, antioxidant activity, industrial processes

## Abstract

Downstream costs represent one of the main obstacles to enabling microalgae to become widespread. The development of an economical, easily scaled-up strategy could reduce the overall process costs. Here, different flocculants were tested on different microalgae strains and a cyanobacterium. The results indicate that flocculation could be an alternative to centrifugation, as CaCl_2_ induced a complete flocculation of green and red marine strains (96 ± 4% and 87.0 ± 0.5%, respectively), whereas Chitosan was the only agent able to induce flocculation on the cyanobacterium (46 ± 1%). As for the thermoacidophilic red microalga, 100% flocculation was achieved only by increasing the pH. Carotenoids were extracted from the flocculated biomass, and the strategy improved with the use of the wet biomass. The results indicate that flocculation does not affect carotenoid yield, which is at least the same than that obtained upon centrifugation and extraction from the wet biomass. Then, for the first time, the biological activity of the extracts obtained from the flocculated biomasses was evaluated. The results indicate that only the green microalga extract shows increased antioxidant activity. In conclusion, this work highlights that a general downstream procedure cannot be developed for microalgae strains but should be rationally tailored.

## 1. Introduction

In the last few years, microalgae biomass has been deeply explored as a sustainable alternative to conventional feedstocks, mainly for the presence of a wide array of bioactive molecules. Indeed, carotenoids, polyunsaturated fatty acids and proteins are all endowed with beneficial properties for human health [1,2]. However, despite microalgae’s enormous potential, large-scale production is still limited by the high cultivation, harvesting and processing costs [3,4]. It has been estimated that the harvesting step could represent up to 30% of the overall downstream costs [5,6], meaning that the optimization of an efficient and economic harvesting procedure is still a challenge. Indeed, the selection of the right technology to harvest the biomass could be a turning point for rendering microalgae exploitation cost-effective at a large scale.

The main drawbacks related to microalgae harvesting are due to cell size, to their overall negative charge and to their low density; for all these reasons, microalgae cells require an economically expensive and energetically intensive dewatering step. To date, the most used harvesting procedures are sedimentation, centrifugation, filtration, flocculation and flotation [7,8,9]. Each procedure presents different advantages and drawbacks, and the choice of technique is usually based on the market value of the molecule to be isolated. Among all the harvesting techniques, filtration and centrifugation are the most effective in terms of biomass recovery [10,11], but they require a high energy input. In the case of filtration, the frequent replacement of filters makes the technique prohibitive at an industrial scale [12,13]. Sedimentation, on the other hand, is an easy and low-cost procedure, but its efficiency strictly depends on culture density, thus making this procedure time-consuming [14]. Flotation and flocculation are the most promising techniques for microalgae biomass harvesting as they are cheap, easy, fast and scalable. Despite its potential, flotation has limited efficiency due to the interaction between cells and air bubbles in the culture medium, as the collapsing of the bubbles could result in flocs breaking [15]. To overcome this limitation, flotation could be performed in the presence of surfactants, but this would increase the overall costs [13]. On the other hand, flocculation is considered the most cost-effective method, as it requires low energy consumption and is suitable with algal scaling-up systems and the costs are mainly related to the flocculating agent chosen [16]. Flocculants based on metal salts, such as AlCl_2_, AlCl_3_, FeCl_3_, Al_2_(SO_4_)_3_ and Fe_2_(SO_4_)_3_, are generally used for their low costs and are commonly used for biofuel production or wastewater treatments, without considering the effects of flocculants on biomass quality or the environment [6,17,18]. However, due to their non-biodegradable nature, these flocculants may irreversibly affect water reuse. Thus, organic flocculants, such as chitosan and starch, have been proposed as green alternatives. Flocculation can also be achieved by increasing the pH of the culture medium (autoflocculation), avoiding the use of flocculants, with low energy consumption [18]. Recently, new approaches based on magnetic particles have emerged, but their economic feasibility and biocompatibility still need to be evaluated [19].

Besides harvesting, the extraction of biologically active molecules from the biomass represents another challenge to be overcome to fully exploit microalgae’s potential [20]. Conventional extractions usually require large amounts of organic solvents, which are considered not eco-friendly, require long times, give low yields and are generally used on dried biomass, with a consequent increase in the final costs [21,22,23]. Recently, some green extraction techniques were developed, such as pressurized/supercritical liquid extractions and ultrasound- or microwave-assisted extractions. However, these procedures still present some drawbacks, such as scaling-up difficulty, energy consumption, the quality and selectivity of the extractions, cost validation and long extraction times [24,25].

Interestingly, many papers have been published providing alternatives for harvesting the biomass, but the literature is quiescent on the effects that the harvesting methods may have on the quality of the biomass and thus on the extracted molecules. Here, different alternatives, based on eco-friendly and cost-effective flocculation procedures, were provided by taking into account different microalgae strains and flocculants. Moreover, evidence of the effect of flocculants on the subsequent carotenoid extractions was provided. The extraction step was improved, avoiding the use of a dried biomass and using a GRAS solvent, and the whole procedure was validated by assaying the in vitro antioxidant activity of carotenoids.

## 2. Results

### 2.1. Flocculation

To obtain a general understanding of the procedure, different microalgae strains were analyzed: two Rhodophyta (*Porphyridium cruentum* and *Galdieria phlegrea*), a Chlorophyta (*Pseudococcomyxa simplex*) and a Cyanobacterium (*Synechococcus bigranulatus*). Different flocculants, based on their mechanism of action, were tested on each strain: a carbohydrate, chitosan, known to induce flocculation through a bridging mechanism; two salts, calcium chloride and sodium glutamate, which should induce charge neutralization; and the increase of the pH of the cell culture, which causes flocculation through a sweeping mechanism. Flocculants were added to the culture, as described in the Materials and Methods section. For each experimental procedure, the culture was collected when the cell concentration reached 7 ± 0.1 O.D./mL, and then each flocculant was added to the culture medium at three different concentrations (or pH values). Solutions were stirred and analyzed immediately (0 min). To verify the effects of incubation time on flocculation efficiency, longer incubations (10 and 20 min) were performed before reading the absorbance of the culture. Samples were analyzed spectrophotometrically, by reading the absorbance at 730 nm. Each sample was compared to an untreated cell culture.

#### 2.1.1. Chitosan

**Figure 1:**
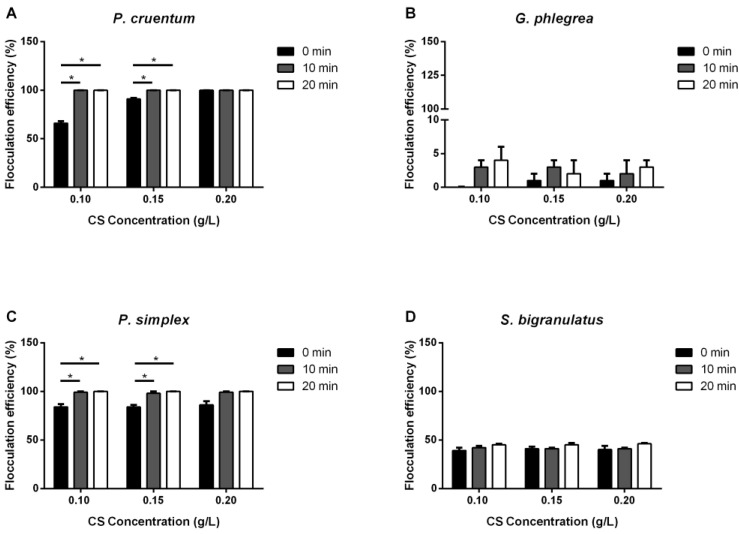
Flocculation efficiency (%) of chitosan (CS). Effect of different CS concentrations on the flocculation efficiency of microalgae strains analyzed immediately (0 min, black bars), 10 min (grey bars) and 20 min (white bars) after stirring. (**A**), *P. cruentum*; (**B**), *G. phlegrea*; (**C**), *P. simplex*; (**D**), *S. bigranulatus* upon different flocculation times. Data are obtained by means of three independent experiments (mean ± S.D.). * *p* < 0.05. The lines above the bars indicate the two samples compared for statistical analysis.

Chitosan (CS) was tested at 0.1, 0.15 and 0.2 g/L. Results are reported in Figure 1. In the case of the *P. cruentum* culture, a direct correlation between the concentration of polymer added and flocculation efficiency was observed: from 66% of flocs immediately formed (0.1 g/L of CS), up to 90% with 0.15 g/L, and 100% flocculation in the presence of the highest concentration tested (0.2 g/L). *P. cruentum* was completely flocculated in the presence of any CS concentration tested after 10 or 20 min. On the other hand, the other red microalga analyzed, *G. phlegrea*, did not flocculate at any time with any tested CS concentration. *G. phlegrea* was incubated up to 90 min in the presence of increasing CS concentrations (0.10–0.20 g/L), but no flocculation occurred. CS induced an immediate 84% flocculation in the green microalga *P. simplex*, independently from the amount of polymer added to the culture. *P. simplex* completely flocculated after 10 or 20 min incubation, independently from the CS concentration. Similar results were obtained with the *S. bigranulatus* culture, even if at a lower extent, as only 40% flocculation was observed independently from the concentration of CS used. It is interesting to note that no significant increase in the efficiency of the process was observed upon 10 or 20 min incubation.

#### 2.1.2. Calcium Chloride and Sodium Glutamate

**Figure 2:**
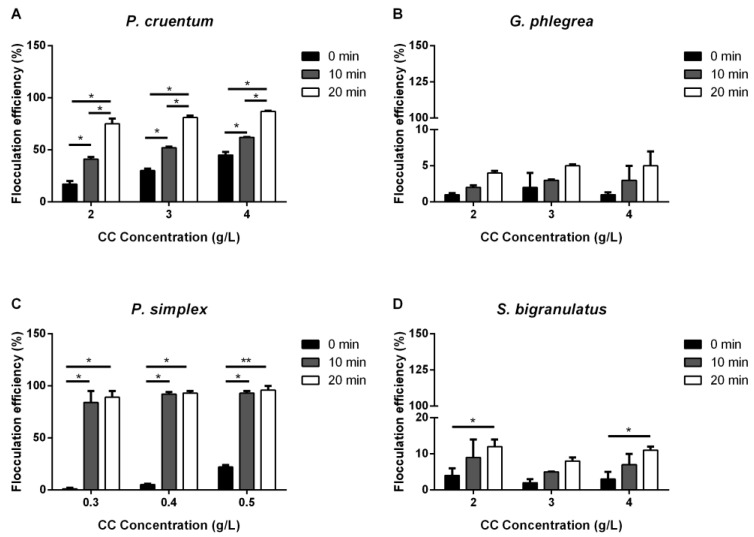
Flocculation efficiency (%) of calcium chloride (CC). Effect of different CC concentrations on the flocculation efficiency of microalgae strains analyzed immediately (0 min, black bars), after 10 min (grey bars) and after 20 min (white bars) from stirring. (**A**), *P. cruentum*; (**B**), *G. phlegrea*; (**C**), *P. simplex*; (**D**), *S. bigranulatus* upon different flocculation times. Data are obtained by means of three independent experiments (mean ± S.D.). * *p* < 0.05, ** *p* < 0.005. The lines above the bars indicate the two samples compared for statistical analysis.

The same set of experiments was carried out using two salts, sodium glutamate (SG) and calcium chloride (CC). SG was tested as a possible cost-effective alternative to polyglutamic acid, normally used in the cosmeceutical industry [26]. When SG was tested, no effect was observed at any concentration used on any strain, independently of the incubation time used. The case of CC was different. As shown in Figure 2, the red microalga *P. cruentum* showed a time- and concentration-dependent flocculation efficiency, reaching about 90% flocculation at the highest CC concentration tested. CC was unable to induce flocculation on the other red microalga (*G. phlegrea*), at any analyzed experimental condition. *P. simplex* flocs were observed after 10 min incubation, with a 90% flocculation efficiency, but independently from the concentration of salt used. No flocculation was observed for *S. bigranulatus* at any analyzed condition.

#### 2.1.3. pH-Mediated Flocculation

**Figure 3:**
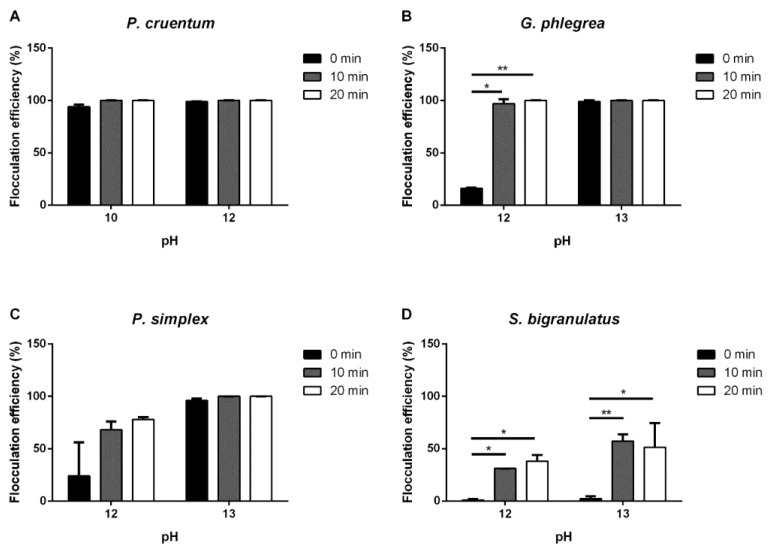
Flocculation efficiency (%) after pH increase. Effect of pH on the flocculation efficiency of microalgae strains analyzed immediately (0 min, black bars), after 10 min (grey bars) and after 20 min (white bars) from stirring. (**A**), *P. cruentum*; (**B**), *G. phlegrea*; (**C**), *P. simplex*; (**D**), *S. bigranulatus* upon different flocculation times. Data are obtained by means of three independent experiments (mean ± S.D.). * *p* < 0.05, ** *p* < 0.005. The lines above the bars indicate the two samples compared for statistical analysis.

Finally, pH increase was tested to analyze autoflocculation. Sodium hydroxide was used to increase the pH of the culture medium, and two pH values were tested for each strain, as indicated in Figure 3. Interestingly, with the exception of the *S. bigranulatus* culture, increasing pH values induced biomass flocculation on all the tested strains. In particular, the increase of the pH value from 7 to 10 immediately induced more than 90% flocculation in the *P. cruentum* culture. This value reached 100% after 10 min incubation. When the pH value was increased from 7 to 12, an immediate and complete flocculation was observed. In the case of the *G. phlegrea* culture, the flocculation efficiency was complete after 10 min incubation. Surprisingly, when the pH of the culture was increased to 13, a complete and immediate flocculation occurred, but a bleaching phenomenon in the biomass happened. Different results were observed for *P. simplex*, in which the switch in the pH value from 8 to 12 induced about 80% flocculation after 20 min incubation, whereas the increase in the pH to 13 sped up the process, and the biomass was totally and immediately flocculated. *S. bigranulatus*, instead, showed a small flocculation efficiency over time at pH 12 (from 1 to 38%) and up to 51% after 20 min at pH 13.

Thus, according to the literature, a high variability of flocculation is observed among microalgae strains, so that each strain represents a different case study [27]. Based on the overall results, for each strain the best flocculation condition was chosen and reported in Table 1. In the case of *P. cruentum*, despite the fact that CS provided the best results, the choice fell on CC, as the latter is a valid and economical alternative (about 1000 vs. 44 €/kg, from Sigma-Aldrich, St Louis, MO, USA).

### 2.2. Pigment Extraction

**Figure 4:**
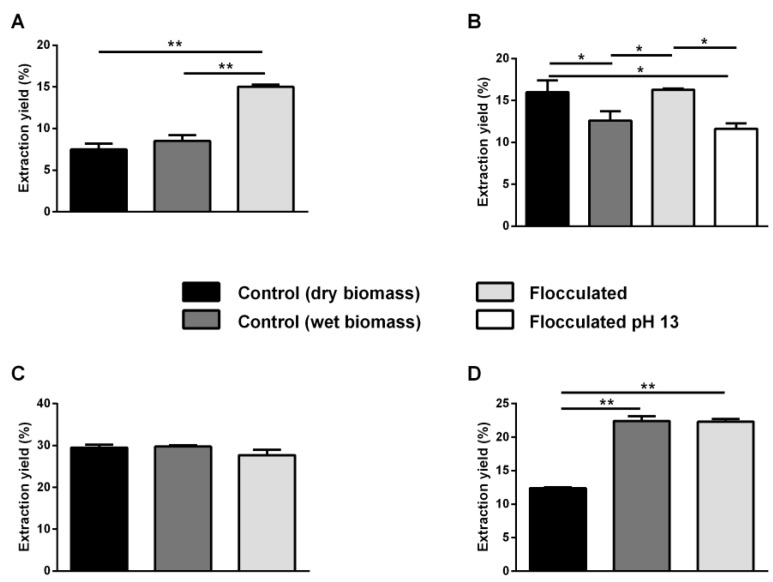
Pigments extraction. Extraction yields (%) are reported as mg of extract with respect to mg of D.W. biomass. Black bars indicate centrifuged dry biomass, dark grey bars indicate centrifuged wet biomass, light grey bars indicate flocculated wet biomass. The white bar refers to flocculated biomass at pH 13. (**A**), *P. cruentum*; (**B**), *G. phlegrea*; (**C**), *P. simplex*; (**D**), *S. bigranulatus*. Results are expressed as the mean ± S.D. of three independent experiments. * indicates *p* < 0.05, ** indicates *p* < 0.01. The lines above the bars indicate the two samples compared for statistical analysis.

In order to verify whether flocculation has any effect on the subsequent pigment extraction in terms of yield and activity, each strain was flocculated based on the selected conditions, and pigments were extracted from the resulting wet biomass. Pigments were extracted by using ethanol as a solvent, as it is GRAS, non-toxic for humans and widely used in many applications. The idea of using wet biomass is another small step forward for the use and application of microalgae, as the drying step is avoided, thus saving energy and reducing the overall costs. Each extraction was compared to two benchmarks, i.e., a biomass harvested by centrifugation and extracted bypassing the drying step (named wet biomass) or after lyophilization (named dry biomass). As described in Materials and Methods, for each extraction, the same amount of biomass was used for pigment extraction by resuspending the biomass in 4 mL of pure ethanol and disrupting by ultrasonication. The volume was then diluted 2.5-fold, in order to increase the efficiency of the extraction procedure, by moving forward the equilibrium between the components still bound to the matrix and those already solubilized in the solvent. In the case of *P. cruentum* (Figure 4A), the yield of the extract obtained from the biomass flocculated with 4 g/L of CC (light grey bar, 15%) showed an increase in flocculation efficiency compared to both untreated samples (black bar, 7.5%, dark grey bar, 8.5%). No significant differences (*p* > 0.05) were observed between the extracts obtained from dry and wet biomass (black bar and dark grey bar, respectively). *G. phlegrea* pigment extraction was performed either on the biomass obtained upon flocculation at pH 12 or on the bleached biomass (obtained at pH 13). As shown in Figure 4B, the yield of the extract obtained from dry biomass (black bar, 16%) was significantly higher than that obtained from the wet biomass (dark grey bar, 13%, *p* < 0.05), whereas no significant difference was observed with respect to the flocculated biomass at pH 12 (light grey bar, 16%, *p* > 0.05). The yield of extraction from the flocculated biomass at pH 12 was significantly higher than that obtained from the wet one (16 and 13%, respectively, *p* < 0.05). In the case of the extract obtained from the flocculated biomass at pH 13 (white bar, 12%), a significant decrease in the extraction yield was observed with respect to dry biomass and to the biomass flocculated at pH 12 (*p* < 0.05). No differences were observed between the extract obtained from the wet biomass and that obtained after flocculation at pH 13 (*p* > 0.05). The extracts obtained from *P. simplex* biomass (Figure 4C) did not show a statistically significant difference among them, suggesting that flocculation does not affect extraction yield. In Figure 4D, the extracts obtained from *S. bigranulatus* biomass are reported. The same yield of extraction (22%) was obtained in the centrifuged wet biomass (dark grey bar) and the flocculated one (light grey bar), and the values were significantly higher (*p* < 0.01) than the yield obtained from the centrifuged dry biomass (black bar, 12%).

### 2.3. In Vitro Antioxidant Activity

**Figure 5:**
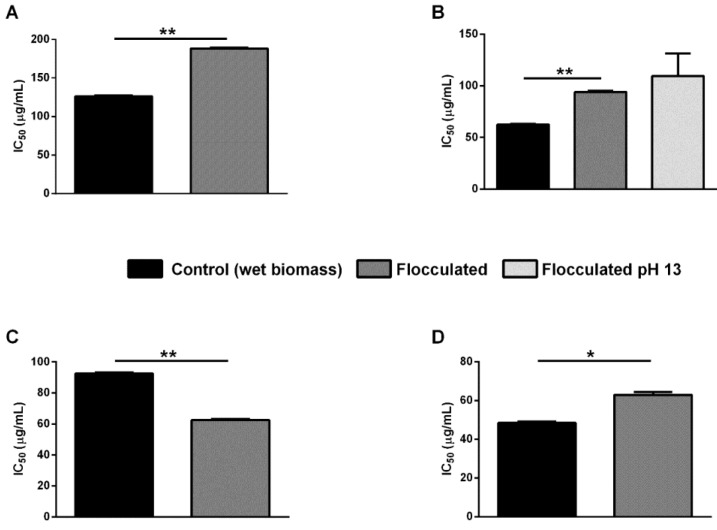
Antioxidant activity of ethanol extracts. Values are expressed as IC_50_ (μg/mL), i.e., the concentration required to scavenge 50% of free radical ABTS. Black bars indicate centrifuged wet biomass, dark grey bars indicate flocculated wet biomass, and the light grey bar indicates flocculated wet biomass at pH 13. (**A**), *P. cruentum*; (**B**), *G. phlegrea*; (**C**), *P. simplex*; (**D**), *S. bigranulatus*. Data are shown as means ± S.D. Three independent measurements were carried out. * indicate *p* < 0.01; ** indicate *p* < 0.001 with respect to centrifuged wet biomass. The lines above the bars indicate the two samples compared for statistical analysis.

From previous data, it is known that the ethanol extract from *P. cruentum* mainly contains zeaxanthin [28], whereas *S. bigranulatus* extract is enriched in zeaxanthin and xanthophylls [29], *G. phlegrea* contains zeaxanthin and β-carotene [30], and *P. simplex* is enriched in lutein [31]. The ethanol extracts, obtained from either centrifuged or flocculated wet biomasses, were analyzed for their antioxidant capacity by in vitro ABTS assay. The results of the ABTS assay, shown in Figure 5, report, for each analyzed condition, the IC_50_ values obtained (i.e., the concentration of extract necessary to inhibit 50% of the free radicals of the ABTS^•+^). In particular, the experiments carried out on the extract obtained from the flocculated biomass of *P. cruentum* revealed a significant increase in the IC_50_ value (187.0 ± 1.1 μg/mL) compared to the extract obtained from centrifuged wet biomass (125.0 ± 1.4 μg/mL, *p* < 0.001). Furthermore, in the case of *G. phlegrea* extract, a significant increase (*p* < 0.001) in the IC_50_ value was observed in the flocculated biomass with respect to the benchmark extraction (i.e., centrifuged wet biomass) (93 ± 1 μg/mL and 63 ± 1 μg/mL, respectively), whereas the extract obtained from the biomass flocculated at pH 13 had an IC_50_ value of and of 109 ± 22 μg/mL (*p* < 0.05 compared to the other tested samples). These results suggest that flocculation can negatively affect the antioxidant activity of the extracts. However, the IC_50_ values obtained from *P. simplex* extracts after flocculation showed a significant reduction in comparison to that obtained from the centrifuged biomass (63 ± 1 μg/mL and 93 ± 1 μg/mL, respectively, *p* < 0.001), thus representing an improvement in the antioxidant activity of the extract. Unfortunately, CS showed a small negative effect on *S. bigranulatus* extract, as the IC_50_ values increased from 48 ± 1 to 63 ± 1 μg/mL (*p* < 0.01). It can be hypothesized that the flocculant may interact with the extracted carotenoid, as CC negatively affects the antioxidant activity of *P. cruentum* (enriched in zeaxanthin) but positively affects the antioxidant activity of *P. simplex* (enriched in lutein).

## 3. Discussion

In the last few years, flocculation has been studied as a cost-effective alternative to centrifugation, with the aim to lower energy consumption and the costs related to the downstream at an industrial level. However, due to the complexity of living systems, such as microalgae and cyanobacteria, it is hard to develop a standardized flocculation procedure that can be effective on different strains [32]. Here, a study on the effect of four flocculants of different natures was carried out on three microalgae strains and one cyanobacterium. Generally speaking, a systematic comparison of different strains could be challenging, as cultivation conditions can differ in some aspects, such as cell concentration or cultivation techniques [27]. For this reason, algae cultures with the same biomass concentrations (7 O.D./mL) were used for all the experiments. Different flocculants were used, and flocculation time was set-up considering the nature of the flocculant as well as the strain analyzed. Typically, an incubation between 0 and 20 min was used, as a prolonged time could overlap with the spontaneous sedimentation that naturally occurs when a culture is in a static mode. Chitosan (CS) was selected as it is a natural and environmentally friendly agent. From a chemical point of view, CS is a polymer obtained from the deacetylation of chitin, and due to its cationic nature, it can interact with negatively charged microorganisms. When the effect of CS was studied on the *P. cruentum* culture, a dose-dependence was observed, as 66% of flocs immediately formed at the lowest concentration tested (0.1 g/L), reaching 100% flocculation in the presence of the highest concentration tested (0.2 g/L). These results indicate a higher flocculation efficiency compared to those reported by Endrawati and colleagues, who found 67% flocculation after 10 min of incubation with 0.1 g/L CS and 100% flocculation only after 40 min [33]. In the case of *G. phlegrea*, no flocculation occurred at any of the experimental conditions tested, whereas CS was able to induce 84% flocculation at time 0 in the green microalga *P. simplex*, independently from the concentration used. Similar results were obtained with *S. bigranulatus*, but at a lower extent (40% flocculation efficiency independently from the concentration). Except for *P. cruentum* flocculated in the presence of CS [33], no data are present in the literature on the other analyzed strains. However, it is generally recognized that in CS-induced flocculation, the incubation time, as well as CS concentration, can affect efficiency. As an example, Ahmad and colleagues reported the flocculation of *Chlorella* sp. in the presence of CS, but the authors found a negative correlation between algal coagulation, the amount of CS used, incubation time and speed rate [34].

Among chemical flocculants, metal ions have been extensively studied for microalgae harvesting, as they are considered highly effective in terms of flocculation efficiency; however, several issues may occur as a possible transfer of the metal ion to microalgae biomass could turn out to be highly toxic [35]. Aluminum sulfate is one of the most used chemical flocculants, generally used for waste water treatment, but it was not used in this study, as it is toxic and its presence would affect biomass valorization [36]. Alternatively, calcium chloride (CC) is a non-toxic alkali metal generally used as a food preservative, and therefore it could represent a green alternative to metal-based salts, to preserve both the biomass integrity and the environment [37]. Indeed, CC was found to be effective on *P. cruentum* in a time- and concentration-dependent manner, whereas *P. simplex* biomass flocculated after 10 min incubation without a dose-effect dependency. Unfortunately, no effects were observed on *G. phlegrea* and *S. bigranulatus*. Overall, the results obtained on the green microalga are consistent with those reported by Singh et al. [37] on *Chlorella pyrenoidosa*. Authors reached almost 98% of flocculation in the presence of~0.3 g/L of CC but with a longer incubation time then that reported in the present paper. Further analyses are needed to verify its effects on cell morphology. Another unexplored chemical flocculant, sodium glutamate (SG), was tested. SG is the monomer of the normally used polyglutamic acid (PGA) [38]. PGA works as a bridging agent by its negatively charged glutamic residue, so it was decided to use the building block to flocculate the biomass, but no positive results were obtained.

Autoflocculation occurs when microorganisms self-aggregate when the environmental conditions change. This phenomenon is achieved by neutralizing the negatively charged cell surface, thus creating an alkaline environment (pH > 8.0) that allows cells to aggregate. The variation in pH can be facilitated by adding to the microalgae culture a small amount of chemicals, such as NaOH, KOH or Mg(OH)_2_, thus making this process economically suitable [39]. However, a substantial increase in pH could affect the algae culture and thus the quality of the biomass [12,40]. Here, NaOH was used to increase the pH of the algae culture, and with the exception of *S. bigranulatus*, it was found to be effective on all the other strains. In particular, for *P. cruentum* and *G. phlegrea* a highly efficient flocculation was immediately observed, whereas *P. simplex* completely flocculated upon 10 min incubation. These results are consistent with the literature data, as an increase in pH values has been shown to induce autoflocculation, with NaOH the base working at lower concentrations with respect to KOH, Mg(OH)_2_ and Al(OH)_2_ [18,41]. Despite the promising results, the pH variation in the case of *G. phlegrea* from 1.5 to 13 resulted in a dramatic change in the color of the biomass, suggesting that flocculation could affect not only the quality of the biomass but also the quality of the molecules within it. This evidence is supported by data in the literature, which report that pH variations can alter microalgae cell physiology [42]. Having selected the best flocculation parameter for each strain, a pigment extraction was carried out. Conventionally, extractions are performed on dry biomasses; however, it is known that the drying step is energetically expensive [30]. To further decrease the overall process costs, in this work pigment extractions were carried out on a wet biomass. The results clearly indicate that flocculation does not negatively affect the extraction yields when compared to those obtained starting from a centrifuged biomass and that the drying step can be avoided, with a generally positive impact on the overall downstream costs. Finally, the bioactivity of the extracted molecules was determined in vitro to understand more closely whether the flocculation procedure could affect the bioactivity of the extracted molecules. The ABTS assay revealed that flocculation does not affect the quality of the extracted molecules, with the exception of *P. simplex*, in which the antioxidant activity of the extract obtained from the flocculated biomass improved with respect to that obtained from the centrifuged one (IC_50_ values: 63 μg/mL vs. 93 μg/mL). Further analyses are mandatory to understand whether an excess of flocculant may be responsible for the lower antioxidant activity of the extract. These results are important as this is the first report in which flocculants have been related to the quality of the extracted molecules. Many papers have been published so far reporting a simple relationship between the efficiency of flocculation and biomass morphology [18,35,36]. However, no analysis on the bioactivity of the molecules extracted from flocculated biomass has been reported so far, probably because research has been mainly focused on wastewater treatment or biodiesel production [37,38,39]. To date, only Taghavijeloudar and colleagues successfully extracted proteins, carbohydrates and lipids from a *Chlorella sorokiniana* strain, but no data are reported on the biological activity of the obtained molecules [43]. Understanding the domino effect of flocculants on biomass and molecules to be extracted represents a priority for a safe and mindful use of microalgae, which are now considered in a wider perspective, not only for their lipid content, but as a source of green, vegan and sustainable molecules.

## 4. Materials and Methods

### 4.1. Reagents and Chemicals

Solvents and reagents, unless differently specified, were from Sigma-Aldrich.

### 4.2. Microalgae Strains and Culture Conditions

*Porphyridium cruentum* (CCALA 415) was acquired from Culture Collection of Autotrophic Organism (CCALA, Centre for Phycology, Institute of Botany of the AS CR, Dukelská 135, TŘEBOŇ CZ-379 82, Czech Republic), and grown in Porphyridium Medium. *Galdieria phlegrea* (ACUF 009), *Pseudococcomyxa simplex* (ACUF 127) and *Synechococcus bigranulatus* (ACUF 680) were kindly provided by Algal Collection of the University Federico II (ACUF, University of Naples Federico II, Department of Biology, Naples, Italy, www.acuf.net, accessed on 5 July 2024) and grown in Allen medium, BBM and BG11, respectively. Microalgae strains were grown in bubble column photobioreactors at temperatures, and light intensity [Photosynthetic Active Radiations (PAR) (μmol_photons_/m^2^/s), as previously described [28,29,31,44]. Briefly, cells were cultured as follows: *P. cruentum* at 25 °C and 13 PAR; *G. phlegrea* at 37 °C and 200 PAR; *P. simplex* at 24 °C and 100 PAR and *S. bigranulatus* at 39 °C and 300 PAR. Each culture was mixed by bubbling air through a sintered glass tube placed at the bottom of each reactor. Algal growth was daily monitored by measuring the absorbance at 730 nm.

### 4.3. Flocculation

At the end of cultivation, different concentrations of high-molecular-weight chitosan (CS), calcium chloride (CC), sodium glutamate (SG) and sodium hydroxide (to increase the pH) were added to each strain. In particular, CS was tested at 0.1, 0.15 and 0.2 g/L; CC at 2, 3, and 4 g/L on *P. cruentum*, *G. phlegrea* and *S. bigranulatus* and at 0.3, 0.4, 0.5 g/L on *P. simplex*. Autoflocculation was induced by adjusting the pH at 10 and 12 for *P. cruentum* and at 12 and 13 for the other strains. Flocculation was carried out at the pH measured at the end of cell growth (7 for *P. cruentum*, 1.5 for *G. phlegrea*, 8 for *S. bigranulatus* and *P. simplex*). After the flocculating agent had been added, each culture was stirred for 30 s at 1000 rpm, and then the O.D. of the solution was measured at 730 nm. Time-course experiments were performed by measuring the O.D. immediately (0 min) and at 10 and 20 min after stirring. Flocculation efficiency was calculated as percentage (%) using the formula:

$$Flocculation\;effiency\left(\%\right)=\left(1-\frac{O.D.sample}{O.D.culture}\times 100\right)$$

where O.D. sample is the O.D. measured after the treatment and O.D. culture is the value measured before any treatment [45].

### 4.4. Pigments Extraction and Yields Determination

Pigment extractions were performed using a GRAS solvent, ethanol, as reported by Aremu, with some modifications [46]. Briefly, for each extraction, independently from the harvesting procedure followed, the equivalent of 200 mg of dry weight (D.W.) was used for extractions. The biomass was suspended in 4 mL of pure ethanol and disrupted by ultrasonication (40% amplitude, 4 min on ice, Bandelin SONOPULS HD 3200, tip MS73). The final volume was adjusted to 20 mL, and the mixture was shaken for 24 h at 250 rpm in a dark room at 4 °C. The mixture was then centrifuged at 5000× *g* for 10 min, and the supernatant was stored at −20 °C and dried under an N_2_ stream at 30 °C. The extraction yields were determined gravimetrically and expressed using the formula:

$$Extraction\;yield\;\left(\%\right)=\;\frac{{mg}_{extract}}{{mg}_{biomass\;dry\;weight}}\times 100$$

### 4.5. ABTS Assay

The in vitro antioxidant activity of each extract was evaluated by the 2,2′-azinobis-(3-ethylbenzothiazoiline-6-sulfonic acid) ABTS assay, according to Imbimbo et al. [30]. Results are expressed as IC_50_ (μg/mL), i.e., the concentration required to scavenge 50% of free radical ABTS.

### 4.6. Statistical Analyses

Samples were tested in three independent analyses, each carried out in triplicate. The results are presented as the mean of results obtained after three independent experiments (mean ± S.D.) and compared by one-way ANOVA according to Bonferroni’s method (post hoc) using Graphpad Prism for Windows, version 6.01.

## 5. Conclusions

In the present work, three microalgae strains and a cyanobacterium were harvested using different flocculants, chosen for their different mechanisms of action. The results clearly indicate that a general procedure cannot be developed, as no correlation between the flocculating agent and the microbial strain can be established, but flocculation must be tailored considering the strain, the nature of the flocculant and the incubation time. It is worth noting that, in this paper, a new carotenoid extraction strategy was set up, based on the use of ethanol on a wet-flocculated biomass. Using a GRAS solvent and avoiding the drying step environmental impact and energy consumption can be reduced, without negatively affecting extraction yields. For the first time, the antioxidant activity of the molecules extracted from a flocculated biomass was analyzed. Unexpectedly, a general decrease in the antioxidant activity was observed on the extracts obtained from the flocculated biomass, except for *P. simplex*, whose activity was increased. Despite the energetic sustainability of the whole process, the presented results raise some criticisms that should be overcome to translate flocculation to an industrial level.

## Figures and Tables

**Table 1:** Summary of the selected flocculation conditions for each strain.

Strain	Flocculation Condition	Flocculation Efficiency 20 Min
*P. cruentum*	4.0 g/L CC	87.0 ± 0.5
*G. phlegrea*	pH 12 pH 13	100.0 ± 0.1 100.0 ± 0.1
*P. simplex*	0.5 g/L CC	96 ± 4
*S. bigranulatus*	0.2 g/L CS	46 ± 1

## Data Availability

Data is contained within the article.
